# Exploring Stakeholder Involvement in Intervention Implementation Studies: Systematic Evidence Synthesis With an Evidence Gap Map Approach

**DOI:** 10.1177/01632787251352837

**Published:** 2025-06-26

**Authors:** Kristina Arnahoutova, Sabina De Geest, Juliane Mielke, Annette Boaz, Helene Schoemans, Sabine Valenta

**Affiliations:** 1 Department of Primary Care and Public Health, Academic Centre for Nursing and Midwifery, 82264 KU Leuven, Leuven, Belgium; 2 Nursing Science, Department of Public Health, 27209 University of Basel, Basel, Switzerland; 3 Health and Social Care Workforce Research Unit, The Policy Institute, King’s College London, London, UK; 4 Department of Hematology, 60182 University Hospitals Leuven, Leuven, Belgium; 5 Practice Development and Research Division, Medical Directorate, 27209 University Hospital Basel, Basel, Switzerland

**Keywords:** implementation science, stakeholder involvement, evidence gap map, research involvement, involvement and engagement, stakeholder engagement

## Abstract

Stakeholder involvement (SI) is essential for effective and sustainable intervention implementation, yet practical guidance is lacking. This study mapped SI use in implementation science studies, identified gaps, and proposed a practical framework for improved SI planning. Using an evidence gap map approach, this study built on Mielke et al.’s (2022) methodology, which identified implementation studies from 2015–2020. The search was updated to include studies from 2021–2023 from PubMed, using the same search strategy and inclusion criteria. Data extraction followed the Guidance for Reporting on Involvement of Patients and the Public reporting checklist. From 10,184 studies, a random sample of 2,005 was screened, adding 162 implementation science studies to Mielke et al.’s 110, totaling 272 studies for SI analysis. SI was reported in 89% of studies, but often lacked depth and strategic planning. Stakeholders were mainly engaged during the preparatory phase. Most studies involved micro- and meso-level stakeholders, rarely including macro-level stakeholders. Few described stakeholder selection or preparation. SI was mostly consultative, via interviews, surveys, and focus groups, with limited active collaboration. SI processes and costs were rarely evaluated. Our findings underscore the need for structured, comprehensive SI planning and offer practical recommendations to strengthen SI efforts in implementation research.

## Background

To address the challenges of translating research into real-world practice, implementation science relies on stakeholder involvement (SI) (Beidas et al., 2022; Neta et al., 2018). Stakeholder involvement can be defined as “an iterative process of actively soliciting the knowledge, experience, judgment and values of stakeholders selected to represent a broad range of direct interests in a particular issue, for the purposes of creating a shared understanding and making relevant, transparent and effective decisions” (Deverka et al., 2012, p. 5). Although the appropriateness of the term ‘stakeholder’ can be questioned (MacLeod, 2022; Rocheleau, nd), we used the definition “individuals, organizations or communities that have a direct interest in the process and outcomes of a project, research or policy endeavor” (Deverka et al., 2012, p. 5). Implementation Science takes a pragmatic approach to studying how best to overcome barriers to implementing interventions in practice (Eccles & Mittman, 2006). From analysing the context to explore and prepare for intervention development or adaptation, to implementation and evaluation of implementation and effectiveness outcomes (Aarons et al., 2011), SI is a key implementation strategy integral to the process of change and essential to study the implementation process.

Stakeholder involvement has become a standard expectation in funding proposals (Neta et al., 2018; Villalobos et al., 2023) due to its benefits across research stages and health contexts, from scaling-up mental health services (Eaton et al., 2011) to implementing prevention and control interventions during public health emergencies (Gilmore et al., 2020). It enhances the acceptability, feasibility, and relevance of research, supporting more sustainable, culturally relevant, and context-sensitive intervention implementation (Osobajo et al., 2023; Shrestha et al., 2022; Wallerstein & Duran, 2010). Engaging (non-research) stakeholders ensures that research addresses unmet needs and insights often missed by researchers alone (Pistollato et al., 2024; Schoemans et al., 2025). Their input can enhance study design (Brett et al., 2014; Maurer et al., 2022), improve recruitment and retention (Crocker et al., 2018), tailor data collection tools (Iezzoni et al., 2017), and clarify complex findings (Maurer et al., 2022). They can help overcome logistical, cultural, and regulatory barriers, ensuring appropriate and effective implementation (Gilmore et al., 2020; Nilsen et al., 2020). Stakeholder involvement helps support clear communication and dissemination, increasing public understanding, awareness, and acceptance of findings (Ball et al., 2019), and it promotes accountability and transparency, fostering trust in research (Esmail et al., 2015; LeClair et al., 2020). Furthermore, SI fosters mutual learning and empowerment by expanding knowledge and skills for both researchers and participants (Ball et al., 2019; Brett et al., 2014; Mathur et al., 2008).

Stakeholder involvement is not a binary concept: it ranges from “informing” to “co-creation”, with a continuum from limited power or decision making ability, up to an equal right and responsibility to impact the project for all participating organizations and/or individuals (Goodman & Sanders Thompson, 2017; Rowe & Frewer, 2005). “Informing” is the lowest level, where information only flows in one direction. Stakeholders have limited power and are only made aware of the project. “Consultation” seeks stakeholder input without making commitment that their voice will carry any weight (Goodman & Sanders Thompson, 2017). “Co-creation” involves active collaboration and shared power throughout the project lifecycle, where information sharing is bidirectional, including feedback on stakeholder contributions (Goodman & Sanders Thompson, 2017; Metz, 2015). The umbrella term “*involvement*” will be used here to cover these various levels.

Various organisations from different countries, including governments, non-profit and patient organisations, pharmaceutical industries and academia, have developed their own SI guidelines or theories, such as the Patient-Centered Outcomes Research Institute (PCORI) Engagement Rubric (Sheridan et al., 2017), the Patients Active in Research and Dialogues for an Improved Generation of Medicines (PARADIGM) patient engagement toolbox (PARADIGM, 2020), the principles for community-based participatory research (Israel et al., 1998), the INVOLVE briefing notes (NIHR, 2021), the ‘7Ps’ of stakeholders (Concannon et al., 2012), and the ‘ladder of participation’ (Arnstein, 1969), to name a few. Yet, the field is still missing an commonly accepted definition of SI so it remains an complex, unclear construct, leading to varied terms such as “patient and public involvement”, “community participation”, “participatory research”, “stakeholder engagement”, “co-creation”, “partnerships”, and “citizen science” (Ramanadhan et al., 2024; Ray Kristin & Miller Elizabeth, 2017). Moreover, while involving stakeholders has increasingly been emphasized, there is a lack of literature on how involvement costs and value are being calculated (Anggraeni et al., 2019).

While these existing theories offer valuable guidance, they lack practical steps to plan and initiate SI (Ray Kristin & Miller Elizabeth, 2017; Staniszewska et al., 2018). A structured and intentional approach to recruiting and involving stakeholders is essential (Boaz et al., 2018). Starting with clearly defining the purpose of SI, encouraging reflection on its scope (Boaz et al., 2018; Bombard et al., 2018; Potthoff et al., 2023). Planned stakeholder identification and recruitment enables research teams to engage diverse stakeholders to shape feasible, context-specific solutions (Leventon et al., 2016). Specifying the level of involvement and selecting appropriate methods (e.g., focus groups, individual feedback) ensures contributions are meaningful and that they match the input needs, stakeholder expertise and capacity for involvement (Concannon et al., 2019; NIHR, 2021). Finally, evaluating SI efforts allows for ongoing improvement, refining SI efforts to the dynamic real-world environments (Esmail et al., 2015). We propose here an overview of the current state of knowledge regarding SI in implementation science and propose a practical guidance to streamline further SI efforts within the field.

### Objective

The overall objective of this work was to quantify the volume of SI and critically review and map current SI characteristics in implementation science studies, identifying gaps and challenges. The review was guided by the following key research questions: What proportion of papers address stakeholder involvement? What are the critical gaps in reporting on the Guidance for Reporting on Involvement of Patients and the Public (GRIPP2) reporting checklist recommended SI concepts (theoretical foundations of SI, stakeholder-related information, methodologies, and evaluation of SI)? For further details on the research questions, see Suppl. Table 4.

## Methods

### Study Design

Since our aim was to identify gaps that need further exploration, without providing an overview of all existing SI evidence, we used an evidence gap map (EGM), i.e., a visual or descriptive summary that presents an overview of relevant evidence to quickly understand the scope of the available evidence on a given topic (Campbell et al., 2023). Building on Mielke et al.’s (2022) methodology, who created an EGM for methodological approaches to contextual analysis (110 articles included), we conducted a sub-analysis of her data focusing on SI and supplemented it with an updated search to include more recent literature (162 articles included).

#### Study Selection and Screening

The articles identified from Mielke et al.’s (2022) study (search from 2015 to 2020) were used and, using the same search string, (Suppl. Table 1), were complemented by an updated search covering papers published in Pubmed between January 2021-2023. A random sample comprising 20% of the articles published in each year was selected using a random number generator. This selection was screened for intervention implementation science studies using the inclusion criteria from Mielke et al. (2022) for consistency; all criteria needed to apply (Suppl. Table 1): (a) peer-reviewed articles or study protocols (b) intervention implementation studies (c) using experimental or quasi-experimental designs (d) testing intervention effectiveness (e) in routine practice, (f) including at least one of Brown et al.’s (2017) interventions, and (g) reporting on the implementation pathway evaluation with qualitative or quantitative information on the process or at least one implementation outcome per Proctor et al. (2011) (Suppl. Table 2). Feasibility studies were included only if they assessed at least one additional implementation outcome. Studies had to be (h) written in English or German (i) with full texts available. For SI, we used the definition of Deverka et al. (2012), considering a broad range of stakeholders at the micro-, meso- and macro-level and a broad range of terms referring to the involvement of stakeholders (Suppl. Table 3). Studies were screened in two phases. In the first step, inclusion criteria from suppl. Table 1 were applied to identify intervention implementation science studies, based on review of titles and abstracts, and if relevant, full text. In step 2, the identified studies (n = 272) were further assessed regarding SI. Screening was independently done by the first (KA) and last author (SV) using the web-based tool “*Rayyan*” (Rayyan). After each step, screening of studies was unblinded and conflicts resolved.

#### Data Extraction to Explore Gaps in SI

Based on our research questions (Suppl. Table 4), a detailed data extraction table was drafted (this information can be obtained as an excel file from the authors). Data extraction was performed based on the GRIPP2 reporting checklist. It was developed to improve the quality, transparency, and consistency of reporting on patient and public involvement in research (Staniszewska et al., 2017). Although GRIPP2 was designed for patient and public involvement, it provides a strong foundation for reporting on broader SI. Since the GRIPP2 checklist is designed for reporting on involvement in a paper, we used its aspects but grouped them under SI categories rather than paper sections. Furthermore, to enrich our data extraction, we have customized the GRIPP2 checklist, focusing on capturing more specific details on SI by introducing essential aspects within existing categories to gather broader information. Nineteen dimensions of SI were coded: Theoretical gaps reported in SI, frameworks, aims; stakeholders, employing the 7Ps framework to delineate stakeholders across 7 stakeholder groups (Concannon et al., 2012; Deverka et al., 2012) and their identification (Concannon et al., 2019), preparation (Brett et al., 2010) what stakeholder had to do; the research phases in which stakeholders were involved (NIHR, 2021), the varying levels of SI (IAP2 Public Participation Spectrum), the methods and compensation for involvement; the aim for evaluating SI (Popay et al., 2014), the outcomes and impacts of SI (Ball et al., 2019; Staley, 2009), the methods for evaluation, reported robustness of SI methods, evaluation of frameworks, and context and process factors influencing SI.

#### Data Analysis and Reporting

General study characteristics were analysed descriptively, calculating frequencies and percentages. For the SI data, we initially conducted a descriptive analysis calculating frequencies and percentages to provide a visual representation of the gaps in SI practices.

## Results

### Screening

The updated PubMed search returned 10,184 records. After selecting the random sample, screening and removing duplicates, 2005 records relevant to implementation science remained. Applying implementation science selection criteria, we added 162 implementation studies to the initial 110 intervention implementation studies previously identified by Mielke et al. (2022), totalling 272 papers for SI data extraction (Suppl. Figure 1).

#### General Characteristics

Most of the extracted articles were study protocols (n = 195, 72%), primarily focusing on community services (n = 99, 36%). Most articles were from North America (n = 108, 39.7%) and used experimental study designs (n = 239, 88%). Among studies with hybrid designs (n = 193, 71%), most were described as Hybrid Type II (n = 36; 19%). SI was identified in 241 (89%) of 272 studies. (Suppl. Table 5)

### Overview of EGM in SI

**Figure 1:**
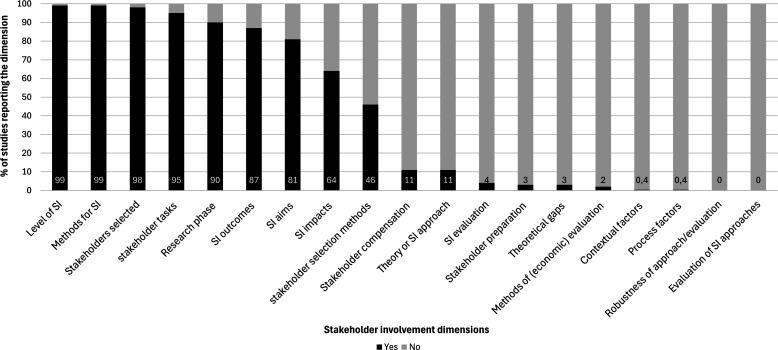
EGM Overview Reporting Frequency of Various Dimensions of SI. *Note.* SI = Stakeholder Involvement

Figure 1 presents an EGM showcasing the reporting frequency of 241 studies across various dimensions of SI, as assessed using the GRIPP2 reporting checklist. Gaps are evident in aspects such as “Evaluation of SI Approaches” (see also Suppl. Table 6).

#### Theoretical Underpinning for SI

Most studies (n = 196, 81%) reported an aim for their SI upfront (Figure 1). Only 27 studies (11%) reported underlying theories for SI efforts (Table 1), like community involvement (4%) or participatory research approaches (3%), which conceptualize the principles of empowerment, collaboration, and shared knowledge, emphasizing the active participation of stakeholders. Eight studies (3%) reported a rationale for why involvement is important (theoretical gaps).

**Table 1:** Applied Theories or SI Approaches to Inform SI and Information on Stakeholders Involved in Intervention Implementation Studies (N = 241)

Identified theories or SI approaches (n = 22)	Reported by studies (n, %)
Reported underlying theoretical foundation or SI approaches:	27 (11%)^a^
**Community involvement approaches** (Banfield et al., 2020; Haynes et al., 2018; Johnson et al., 2018; Levison et al., 2023; Litchman et al., 2022; Liu et al., 2021; McGrath et al., 2021; Nahar et al., 2020; Richards et al., 2021; van Dongen et al., 2019)	10 (4%)
**Participatory research approaches** (Hartman et al., 2021; Kohrt et al., 2022; Kwan et al., 2020; Lakerveld et al., 2018; Lander et al., 2020; McKay et al., 2018; Seguin-Fowler et al., 2022; van Dongen et al., 2019)	8 (3%)
**Citizen engagement approaches** (Seguin-Fowler et al., 2022; Smit et al., 2022)	2 (1%)
**Other theoretical foundation or methodological approach:**	11 (5%)
(Experience-based) Co-design (Lander et al., 2020; Shanley et al., 2019)	2 (1%)
Collaborative intervention planning framework (Markle-Reid et al., 2020; McAiney et al., 2022)	2 (1%)
Team science approach (Smeltzer et al., 2018)	1 (0.4%)
Action cycle (Shanley et al., 2019)	1 (0.4%)
Collaborative participation (Apers et al., 2020)	1 (0.4%)
Darthmouth model for co-design and implementation (Perry et al., 2022)	1 (0.4%)
Patient-centered outcomes research institute engagement rubric (Kearney et al., 2021)	1 (0.4%)
Co-creation approach: Cooperative planning (Grüne et al., 2022)	1 (0.4%)
Normalisation process theory (Clark et al., 2022)	1 (0.4%)
Not reported/unclear	214 (89%)

Stakeholder type	Reported by studies (n, %)
Reported on stakeholders involved:	237 (98%)^b^
Micro-level	
Target population	176 (73%)
Family/caregivers	67 (28%)
Meso-level	
Healthcare stakeholders (provider/institution)	185 (77%)
Public/community	73 (30%)
Other researchers	36 (15%)
Macro-level	
Policy maker/(local) government or leadership	40 (17%)
Payers/funders	12 (5%)
Purchaser	1 (0.4%)
Other	51 (21%)
Not reported/unclear	4 (2%)

Stakeholder selection	Reported by studies (n, %)
Reported on methods for selection:	110 (46%)^c^
Purposive sampling	77 (32%)
Convenience sampling	28 (12%)
Random sampling	25 (10%)
Snowball sampling	10 (4%)
Not reported/unclear	131 (54%)

Preparation for involvement	Reported by studies (n, %)
Reported on preparation:	7 (3%)^d^
Provide project information to stakeholders	3 (1%)
Photovoice training for stakeholders	2 (1%)
Skills learning for stakeholders	1 (0.4%)
Developing cultural competence research team	1 (0.4%)
Not reported/unclear	234 (97%)

*Notes.* ^a^Several studies reported multiple theories, resulting in 22 theories identified in 27 studies.

^b^Several studies reported several stakeholders.

^c^Several studies reported several recruitment techniques.

^d^Several studies reported several forms of preparing stakeholders, 1 study reported stakeholder preparation but did not specify.

#### Identifying Stakeholders and Preparing for Involvement

Authors described involving diverse stakeholders, predominantly micro- (e.g., target population, family/caregivers) and meso-level stakeholders (e.g., clinicians, healthcare administrators, community, and researchers). Macro-level stakeholders (e.g., payers, policymakers) were less reported. Purposive sampling was the predominant method for identifying stakeholders (n = 77, 32%). Only seven studies (3%) addressed stakeholder preparation before involvement (Hodgins et al., 2022; Hoskins et al., 2022; Kearney et al., 2021; Kohrt et al., 2022; Maxwell et al., 2016; Perry et al., 2022; Pevnick et al., 2021; Quintiliani et al., 2015; Shanley et al., 2019). Of these, three studies prepared stakeholders by providing information beforehand. Two studies prepared stakeholders for photovoice methods by familiarizing them with camera usage. One study provided skill-based training, such as public speaking. One study highlighted the research team’s preparation for involving Indigenous communities through cultural competence development (Table 1).

#### Methods for Conducting SI

Table 2 illustrates varying levels of SI across different research phases. Most studies involved stakeholders during the preparatory (52%), intervention (50%) or after trial phase (45%). There was little SI reported at baseline (n = 47, 17%) and during dissemination (n = 34, 14%).

**Table 2:** Methods for Conducting and Evaluating SI in Intervention Implementation Studies (N = 241)

Research phase	Reported by studies (n, % of 241)
Reported on research phase:	217 (90%)^a^
Preparatory phase	126 (52%)
Baseline	47 (17%)
During the intervention trial	120 (50%)
After the intervention trial	110 (45%)
Process evaluation	69 (29%)
Dissemination of findings	34 (14%)
Not reported/unclear	24 (10%)

Level of involvement	Reported by studies (n, % of 241)
Reported on level:	238 (99%)^b^
Consultation (asking for their views)	213 (88%)
Collaboration/co-creation (working together throughout the research process)	103 (43%)
Informing (outreach and education)	32 (13%)
Stakeholder-led (research initiative and decision-making lies with stakeholders)	3 (1%)
Not reported/unclear	3 (1%)

Methods for SI	Reported by studies (n, % of 241)
Reported on methods:	238 (99%)^c^
Interviews	158 (65%)
Surveys	86 (36%)
Focus groups	73 (30%)
Meetings	36 (15%)
Steering/working committee	25 (10%)
Workshops/sessions	25 (10%)
(Community) advisory board	23 (10%)
Observations, notes/reports, logbooks	13 (5%)
Dissemination methods (social media, press release, presentations)	8 (3%)
Discussion groups	6 (3%)
Photovoice	5 (2%)
Jury panel	4 (2%)
Other	22 (9%)
Not reported/unclear	3 (1%)

SI evaluation	Reported by studies (n, % of 241)
Reported on aim SI evaluation:	10 (4%)
Formative assessment (to improve engagement processes)	1 (0.4%)
Process assessment (to identify factors affecting the engagement process)	1 (0.4%)
Summative assessment (to demonstrate the outcomes of the engagement process)	8 (3%)
Not reported/unclear	231 (96%)

Impact of SI	Reported by studies (n, % of 241)
Reported on SI impact:	154 (64%)^d^
Impact on aspects of the research project	123 (51%)
Impact on the stakeholders engaged	5 (2%)
Impact on the research team	3 (1%)
Wider impact	38 (16%)
Economic impact (costs related to SI)	9 (4%)
Not reported/unclear	87 (36%)

*Notes.* SI = stakeholder involvement.

^a^Several studies reported involvement in multiple research phases.

^b^Several studies reported several levels of involvement.

^c^Several studies reported several kinds of methods.

^d^Several studies reported several impacts.

The majority of studies (n = 213, 88%) involved stakeholders through consultation and only three authors (Kearney et al., 2021; Weinert et al., 2022; Wenzel et al., 2021) reported on stakeholder-led research. Tasks assigned to stakeholders were predominantly related to key informant data collection methods such as interviews (n = 158, 65%), focus groups (n = 73, 30%), and surveys (n = 86, 36%) to assess intervention experiences, barriers, facilitators, and implementation outcomes. Eleven percent (n = 27) of studies reported compensating their stakeholders for their input.

#### Methods for Evaluation of SI

Only ten studies (4%) reported evaluating SI, with few detailing their evaluation methods (Table 2). The main evaluation of SI reported related to its impact on the research project itself, such as its effect on intervention development or adaptation (De Geest et al., 2022). The impact of SI on the stakeholders themselves was noted in five studies (2%) (Kearney et al., 2021; Knight et al., 2016; Lander et al., 2020; Marcussen et al., 2022; Quintiliani et al., 2015) while three studies (1%) reported impact of SI on the research team (Fernandez Turienzo et al., 2023; Kearney et al., 2021; Marcussen et al., 2022). Broader impacts of SI were mentioned in 38 studies (16%). Economic impacts of SI were discussed in nine studies (4%), but only two studies described their evaluation methods (Bartakova et al., 2022; Seguin-Fowler et al., 2022). No study reflected on the theoretical underpinning or methods developed for conducting/evaluating SI. Two studies evaluated the external influence of (contextual, n = 1 and process, n = 1) factors on the conducted SI (Adu et al., 2021; Hoskins et al., 2022).

## Discussion

Following the EGM methodology of Mielke et al. (2022), we sought to provide an overview to quickly understand the scope of the available evidence regarding SI in implementation studies. Across the 272 identified implementation science studies, 89% reported some form of SI, which was not surprising given the rising trend of SI in research (Villalobos et al., 2023). However, very few studies reported details regarding theoretical background, methodological aspects, and evaluation of SI, possibly due to a lack of skills and knowledge among scientists regarding SI (Shea et al., 2017), and the absence of SI principles in implementation theories (Villalobos et al., 2023).

### Growing Field of Implementation Science

Compared to Mielke et al.’s (2022) study, encompassing 110 implementation science studies over five years, our updated analysis added 162 studies within a two-year timeframe, a 147% increase in publication rate. This growth underscores the rapid global expansion of this field (Rubin, 2023), and reflects advancements in research methodologies promoting real-world adoption of health innovations and ensuring their long-term sustainability (Blevins et al., 2010; Rubin, 2023).

### SI in Implementation Science

#### Lack of Theory Application and SI Planning

While several theories and approaches for SI exist (Greenhalgh et al., 2019), only 11% of our included studies reported using a guiding theory for their SI efforts (Table 1). These theories broadly fell into three categories: (a) community-based approaches prioritizing partnerships with communities to address research questions (N. B. Wallerstein & Duran, 2006), (b) participatory research approaches focusing on engaging stakeholders to drive social change (Cornish et al., 2023), and (c) citizen involvement approaches that include the broader public in scientific research (Canadian Institute of Health Research, 2012). A few lesser-known models were identified, such as the Darthmouth model for co-design and implementation (Perry et al., 2022) and cooperative planning (Grüne et al., 2022). Despite lacking comprehensive step-by-step guidance, they still provide a structure to help ensure that key elements of SI are considered (Concannon et al., 2012).

Although 81% of studies outlined aims for SI, most focused narrowly on consulting stakeholders about implementation barriers and facilitators. Few studies articulated their specific goals for SI, or explained how stakeholder input would shape research decisions. For example, Grüne et al. (2022) involved stakeholders to identify relevant determinants of physical activity, aiming to enhance intervention’s acceptability. Clearly defining the objective of SI is essential, as it helps shape the scope of SI efforts (Boaz et al., 2018; Bombard et al., 2018; Potthoff et al., 2023). However, involving stakeholders can present challenges, such as power imbalances, language barriers, differing beliefs, and limited time, that should be addressed upfront (Shippee et al., 2015; Shrestha et al., 2022). Without thoughtful planning, SI effort risk resulting in misalignment in expectations, superficial involvement and ethical concerns about the transparency and value of stakeholder contributions (Hahn et al., 2017; Lindsay et al., 2015; Snape et al., 2014; Veisi et al., 2022; Wilkins, 2018).

#### Variability in Stakeholder Identification and Preparation

Our findings reveal a gap in reporting identification and selection of stakeholders. Some studies reported using techniques like convenience and purposive sampling, as described in the literature (Domecq et al., 2014). However, the majority of studies did not specify how stakeholders were identified, nor the criteria used for their selection and recruitment. This lack of detail contrasts with recommendations, which emphasize identifying stakeholders based on their relevance, influence, and interest in the research to ensure diverse perspectives and expertise are represented (Brugha & Varvasovszky, 2000; Concannon et al., 2019). Effective recruitment of stakeholders should involve clear communication to show the mutual benefits of their involvement. This would help build trust, encourage involvement and sustain it over time (Holifield & Williams, 2019).

In clinical research, stakeholders are typically patients and health care providers (Nilsen et al., 2020). However, implementation science requires involving a broader spectrum of stakeholders at the micro-, meso- and macro-level of the system (Brookman-Frazee et al., 2016; Grimshaw et al., 2012; Neta et al., 2018). While we identified substantial involvement of micro- and meso-level stakeholders, macro-level stakeholders, such as payers and policymakers, were rarely mentioned, consistent with findings in implementation grants (Villalobos et al., 2023). These stakeholders should not be overlooked as they play a crucial role in shaping the implementation environment and translating research findings to policy and practice, thereby impacting sustainability (Concannon et al., 2014).

Few studies explicitly addressed how stakeholders or research teams were prepared for involvement, suggesting that the importance of preparatory steps might be underrecognized and/or underreported in the literature. However, preparation is important for building and maintaining trust between stakeholders and researchers throughout the SI process (Boaz et al., 2018; Bombard et al., 2018). Only Shanley et al. (2019) highlighted the importance of preparing research teams, specifically by developing cultural competence and building relationships with stakeholders to increase trust and mutual understanding. Both stakeholders and researchers can benefit from support and training: stakeholders need guidance on the research process (Concannon et al., 2014; Staniszewska et al., 2018), while researchers should receive training on SI (Hubbard et al., 2007). Programs, such as those organized by EUPATI ((EUPATI) https://eupati.eu/eupati-fundamentals/) for instance, provide education and resources to empower patients and stakeholders to be involved in medicines research and development.

#### Lack of SI within Implementation Phases

Reporting on SI varied across research stages, with the highest involvement seen during the preparatory phase. However, SI should ideally be integrated across all phases of implementation projects (Metz & Boaz, 2016; Ramanadhan et al., 2024). In the preparatory phase, stakeholders can play a key role by helping identify and select relevant context factors for analysis, ensuring the work is grounded in practical needs and local insights (Landes et al., 2019; Moullin et al., 2020). They can also contribute to developing or adapting interventions by interpreting findings, and support implementation by helping to select appropriate strategies (Pérez Jolles et al., 2022; Powell et al., 2017; Ramanadhan et al., 2024). Involvement during evaluation phases is also important, as it helps extend outcomes and supports sustainable implementation (Curran et al., 2022; Ramanadhan et al., 2024; Villalobos et al., 2023). Limited reporting of SI in later stages may reflect a bias in literature, with studies focusing more on trial design and less on subsequent phases.

Interestingly, less than half of the studies reported active collaboration with stakeholders. While continuous collaboration or stakeholder-led research may not always be necessary or feasible, deeper and more frequent SI is preferred to fully benefit from stakeholder perspectives and enhance the research impact (Villalobos et al., 2023). Methods such as focus groups, interviews, surveys, are commonly used for SI, reflecting a reliance on traditional forms of consultation (Bombard et al., 2018; Concannon et al., 2014; Domecq et al., 2014). However, these approaches often position stakeholders more as information sources, serving as research participants rather than active collaborators (Villalobos et al., 2023). The limited level of active involvement may stem from the substantial time, resources flexibility required, demanding substantial commitment from researchers (Brett et al., 2014; Concannon et al., 2014).

#### Lack of SI Evaluation and Reporting

There is a notable lack of evaluation of SI, both within our EGM (4%) and in the broader literature (Brett et al., 2014; Price et al., 2018; Villalobos et al., 2023). Despite progress in SI, the evaluation of SI lags behind. Without proper evaluation and reporting, it is challenging to determine whether SI is simply a box-ticking exercise or making a meaningful contributor to the project’s success, and how future SI efforts can be improved (Staley, 2015). The limited availability of conceptual frameworks and validated measurement tools is a key barrier (Ball et al., 2019; Brett et al., 2010; Staniszewska et al., 2018). A few tools have been developed to support this process, such as the Public Involvement Impact Assessment Framework (PIIAF) (Popay et al., 2014) and the Public and Patient Engagement Evaluation Tool (PPEET) (Abelson et al., 2019). Combining these tools with a mixed methods approach can offer a comprehensive understanding of the value of SI (Marshall et al., 2024).

Terminology used in evaluating SI can be ambiguous, with terms like “impact” and “outcomes” often used interchangeably (Brett et al., 2010). Yet, they refer to different aspects of SI effectiveness. Outcomes are specific endpoints of a study resulting from SI efforts and were reported by 86% of our identified studies. Examples include identifying stakeholder needs, satisfaction levels, barriers and facilitators to implementation, and feedback on interventions or implementation strategies. However, many studies fail to report how these outcomes are used or what influence they have, with 63% of identified studies reporting the SI impact. Impact refers to the influence of SI. As noted by Brett et al. (2010), SI can impact various areas: the research process (e.g., refining interventions based on end-user needs (Steele Gray et al., 2016), improving implementation through tailored action plans (Hoskins et al., 2022), and disseminating research findings more broadly (Lander et al., 2020)); stakeholders involved (e.g., personal reflection (Quintiliani et al., 2015), knowledge and skill development (Lander et al., 2020); researcher teams (e.g., gaining insights into patient experiences (Marcussen et al., 2022)); and wider society (e.g., community trust in research, widespread integration of interventions, awareness, and influencing policy changes (Afolabi et al., 2022; Barnes et al., 2022; De Geest et al., 2022).

We observed minimal consideration of how context or process factors influence (<1%). Context is the environment and conditions under which SI occurs (e.g., policy, funding). Process factors relate to how involvement is carried out including the level of involvement, timing and design (Brett et al., 2010). Neglecting these factors can limit the potential impact of SI (Brett et al., 2010). This again highlights the importance of an well-planned, strategic approach to SI (Boaz et al., 2018).

Reporting of costs associated with SI was also typically missing, with only 4% reporting an economic outcome, and less than 1 % specifying methods for its evaluation (Bartakova et al., 2022; Seguin-Fowler et al., 2022). Economic evaluations of SI are generally lacking in the literature (Brett et al., 2010), despite potential impacts on project costs. This could stem from a lack of practices, for example compensating stakeholders for their time and expertise was reported in only 11% studies. Economic evaluations are also interesting for macro-level stakeholders such as funders and policymakers who need to assess cost against benefits (Brett et al., 2010).

### Limitations

Despite our best efforts, we acknowledge several limitations to our work. First, our approach involved limited screening of the available literature, which could potentially result in missing important information from other studies. Additionally, we were limited by time and resources and therefore limited the scope to peer reviewed literature. Stakeholder involvement information was often scattered and poorly described, making it challenging to identify specific details. It is also possible that research projects involved stakeholders without reporting it, as this is not a standard reporting item for implementation studies. Moreover, most papers were protocol papers, which may only reflect aspirations and not actual practices. Further, our analysis does not clarify whether the absence of SI reporting in different research phases was due to the phases not being part of the study or simply SI being undocumented, preventing us from making this distinction. Due to restricted resources, we were unable to conduct any cross-gap analyses, however, this could be considered in future work. Finally, despite our thorough data extraction process, it remains subject to human judgment due to the current heterogeneity of the SI-terminology and definitions.

### Recommendations

We recommend researchers to use SI resources that exist such as the GRIPP2 checklist and systematically reporting on key aspects and challenges linked to SI when publishing on implementation science projects. Systematically embedding SI requirements to implementation science research calls and funding schemes could further advance and normalize SI (Villalobos et al., 2023). Furthermore, there is a need to build an evidence base for evaluating SI, including defining appropriate outcomes and impacts, through systematic reviews on SI evaluation and mixed-methods studies on experiences and impacts.

Regarding conducting SI, researchers should prioritize integrating SI throughout the research process, and develop a comprehensive SI plan tailored to their research project, context, and resources (Boaz et al., 2018; Concannon et al., 2019). Drawing on previous literature and our EGM findings, we have developed recommendations to facilitate the planning of SI in implementation science studies (Table 3 and Suppl. Figure 2): we recommend engaging diverse stakeholders throughout the research project, using varied approaches such as workshops, advisory panels or committees, beyond focus groups or surveys. Researchers should evaluate SI by measuring the process and the impact using mixed methods and incorporate flexibility for continuous review and refinement (Boaz et al., 2018; Concannon et al., 2019; Domecq et al., 2014; Villalobos et al., 2023).

**Table 3:** Stakeholder Involvement Plan Encompassing Critical Aspects of SI

First steps at the beginning of an implementation science-SI project:			
✓ Use a (reporting) theory/framework to ensure critical aspects are covered and reported: GRIPP2 checklist (Staniszewska et al., 2017)			
✓ Conceptualize SI according to EPIS-framework: exploration, preparation, implementation, sustainment (see below) (Moullin et al., 2019)			
✓ Use 7Ps framework: environmental scan to list all stakeholders and their interactions with the broader context or setting (Concannon et al., 2012; Deverka et al., 2012)			
Implementation science phase A		Implementation science phase B	
**Exploration:** plan involvement	**Preparation:** prepare SI With stakeholders	**Implementation:** conduct involvement	**Sustainment**: evaluate and reflect on involvement
✓ Define the **purpose and desired outcomes** by SI (Concannon et al., 2019)✓ **Stakeholder analysis**: Map stakeholders, narrowing down the list to stakeholders important for each research phase (reasons/importance) (Barkhordarian et al., 2015; Brugha & Varvasovszky, 2000; Parker et al., 2022; Smits et al., 2020)✓ Prepare the **involvement plan** (Concannon et al., 2019) ○ Determine **research activities** ○ Determine **level of involvement** for each research activity and each stakeholder (group): Informing (outreach and education), consulting, co-creation or stakeholder-led (Ball et al., 2019; Goodman & Sanders Thompson, 2017; NIHR, 2021; Vaughn & Jacquez, 2020) ○ Define **expectations and responsibilities**, type of work for stakeholders and specify SI methods and approaches to be applied (Engage2020, 2015; Sheridan et al., 2017) ✓ Consider **resources** for SI (budget, time, personnel, training opportunities, contracts) (NIHR, 2021; PARADIGM, 2020)	✓ **Recruit stakeholders** and prepare for involvement (introduction session or materials, share and discuss the contract, provide training if needed, …) (EUPATI, nd; NIHR, 2022) ✓ Establish **communication channels and build trust** by continuous communication and feedback	✓ Gather, analyze, and incorporate stakeholder input into the research as described in the involvement plan ✓ **Document and monitor** SI efforts ✓ **Provide feedback** to stakeholders on the impact of their input on the project (Mathie et al., 2018)	✓ **Evaluate SI efforts** using validated tools or different mixed methods focusing on: (Abelson et al., 2019; Center of Excellence on Partnership with Patients and the public, nd; PARADIGM, 2020; Popay et al., 2014) ○ Impact on the research, on the stakeholders themselves and on the research team ○ SI process (e.g., what worked well/challenging, areas for improvement, appropriateness methods) ✓ Document and **report on the SI process**, outcomes, and impact (Staniszewska et al., 2017) ✓ **Reflect and refine** future involvement efforts if appropriate

*Note.* The table summarizes recommendations for planning and conducting SI in implementation science studies, providing researchers with a structured approach at each phase, drawing on established principles or tools. The table is designed as a practical reference for researchers, providing actions and resources that can be adapted to their context and needs. SI = stakeholder involvement.

## Conclusion

Significant gaps exists in SI within intervention implementation science studies. Despite the increase in studies conducting SI, even in “engaged implementation research,” scientists typically lead projects, with stakeholders minimally and superficially involved, often turning the SI process into a box-ticking exercise (Blevins et al., 2010; Potthoff et al., 2023). Few studies adhere to a structured framework for SI, resulting in further gaps, such as overlooking methods to identify and prepare stakeholders. Furthermore, researchers should strive to improve transparency and clarity in SI, providing detailed descriptions rather than scattering information throughout the text to enhance the accessibility and usability of SI methods and findings (Mathie et al., 2018). Although summarizing all SI evidence is challenging due to its breadth and diversity, we offer recommendations to guide SI planning in implementation science studies.

## Supplemental Material

Supplemental Material - Exploring Stakeholder Involvement in Intervention Implementation Studies: Systematic Evidence Synthesis With an Evidence Gap Map Approach Supplemental Material for Exploring Stakeholder Involvement in Intervention Implementation Studies: Systematic Evidence Synthesis With an Evidence Gap Map Approach by Kristina Arnahoutova, Sabina De Geest, Juliane Mielke, Annette Boaz, Helene Schoemans, and Sabine Valenta in Evaluation & the Health Professions.
