# Supplementary material for: Exploring Stakeholder Involvement in Intervention Implementation Studies: Systematic Evidence Synthesis With an Evidence Gap Map Approach
Source: Eval Health Prof. 2025 Jun 26;49(2):123–39. doi: 10.1177/01632787251352837 (PMC13069137; doi:10.1177/01632787251352837)
Supplement: Supplemental Material - Exploring Stakeholder Involvement in Intervention Implementation Studies: Systematic Evidence Synthesis With an Evidence Gap Map Approach [file sj-pdf-1-ehp-10.1177_01632787251352837.pdf]

Supplementary material

**Table of content**	
**Figure 1.** *Prisma Flow Chart*	p. 2
**Figure 2.** *Bridging SI and implementation science research*	p. 3
**Table 1.** *Search string and eligibility criteria as defined by Mielke et al. (2022) for the identification of implementation studies*	p. 4
**Table 2.** *Implementation outcomes defined by Proctor et al., 2011*	p. 5
**Table 3.** *Stakeholder involvement screening terms for the identification of papers reporting on stakeholder involvement*	p. 6-10
**Table 4.** *Stakeholder involvement categories from the GRIPP2 checklist and adapted research questions for this review*	p. 11
**Table 5.** *Characteristics of all implementation studies included in step 1 (n=272) and SI studies in step 2 (n=241)*	p. 12
**Table 6 .** *Data on Stakeholder involvement summarized from the data extraction table*	p. 13

Figure 1

Prisma Flow Chart

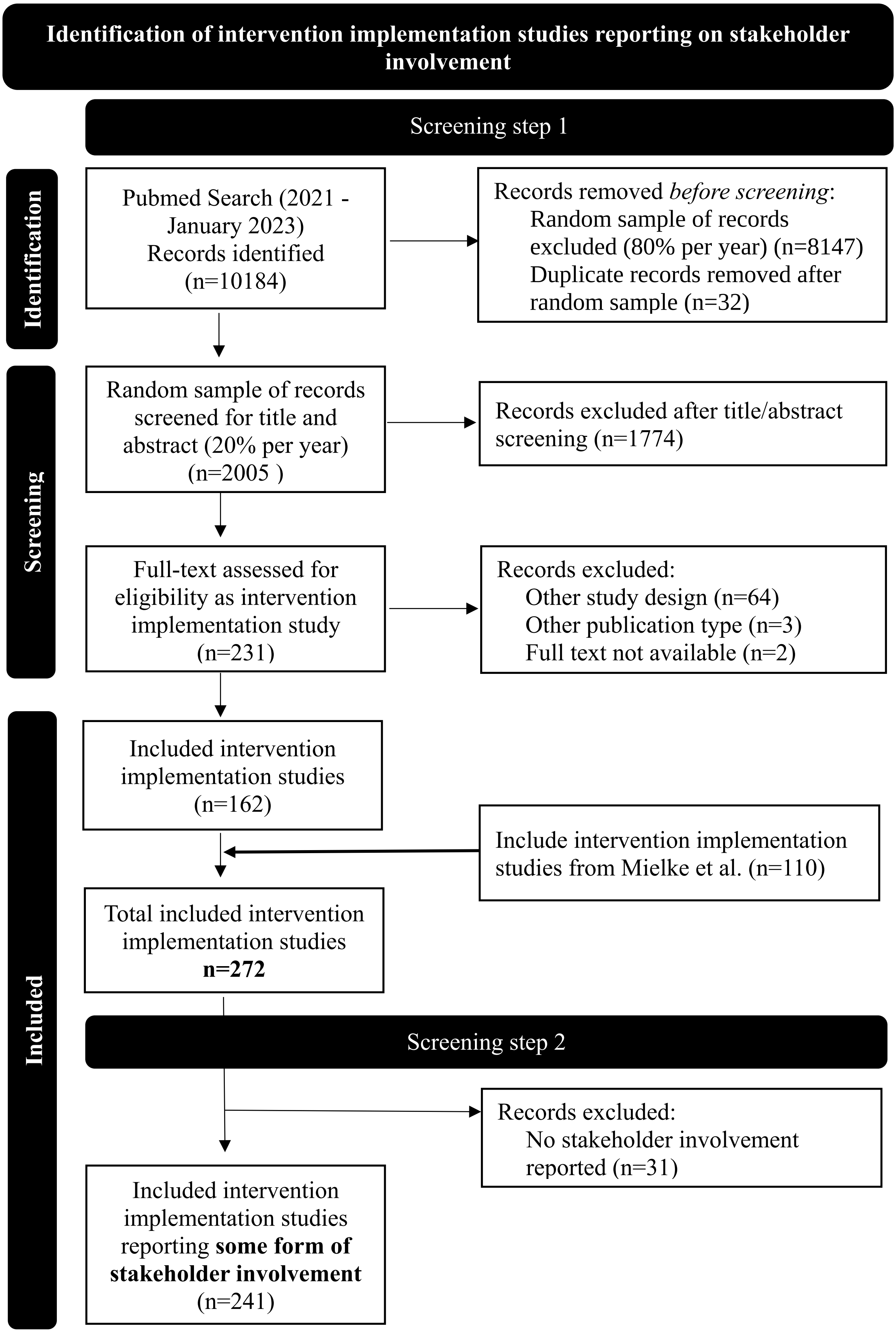

Figure 2

Bridging SI and implementation science research

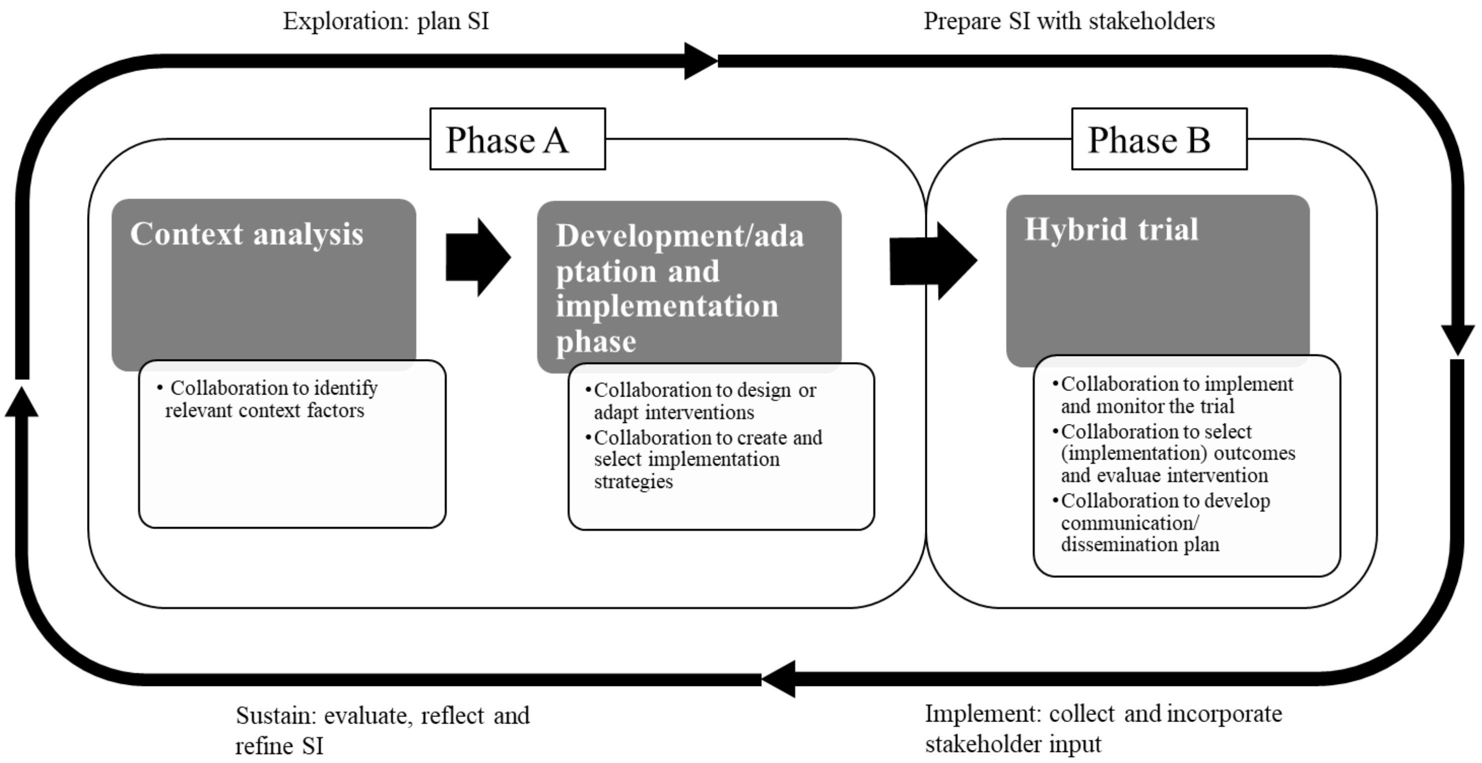

Table 1

Search string and eligibility criteria as defined by Mielke et al. (2022) for the identification of implementation studies

**Search string**<br>implementation science[MeSH Terms] OR implement[Title/Abstract] OR implementation[Title/Abstract] OR introduce[Title/Abstract] OR introduced[Title/Abstract] OR introducing[Title/Abstract] OR introduction[Title/Abstract] **AND** sustainable[Title/Abstract] OR sustainability[Title/Abstract] OR dissemine[Title/Abstract] OR dissemination[Title/Abstract] OR adherent[Title/Abstract] OR adherence[Title/Abstract] OR acceptable[Title/Abstract] OR acceptability[Title/Abstract] OR feasible[Title/Abstract] OR feasibility[Title/Abstract] OR feasibly[Title/Abstract] OR effectiveness[Title/Abstract] **AND** trial[Title/Abstract] OR trialing[Title/Abstract] OR trials[Title/Abstract]	
**Eligibility Criteria - all criteria need to apply**	
**Inclusion criteria**	
Article type<br>Peer-reviewed article or study protocol	Editorials, letters to the editor, commentaries, guidelines, conference abstracts, case reports and dissertation will be excluded.
Study design<br>Implementation intervention study	- Study tests effectiveness of an intervention (experimental or quasi-experimental design).<br>- Intervention tested entails one of the 7 Ps (programs, practices, principles, procedures, products, pills, and policies) [2].<br>- Intervention tested will be implemented in daily (clinical) practice.<br>- Study reports on evaluation of implementation pathway (gathering information on implementation process qualitatively or quantitatively) and/or assessing implementation outcomes as defined by outcomes of Proctor et al. [3]). If feasibility is reported as implementation outcome, at least one further implementation outcome (e.g., acceptability) needs to be reported.
Language	Paper is written in English or German.
Full text	Full text is available.
**Exclusion criteria**	
Process evaluation	Article is clearly labeled as process evaluation (title/abstract).
Study protocol of an included study	If study protocol: The study belonging to the protocol has already been included.

Table 2

Implementation outcomes defined by Proctor et al., 2011

**Implementation outcome**	**Definition** (Gerke et al., 2017)	**Related terms**
Acceptability	Extent to which implementation stakeholders perceive a treatment, service, practice, or innovation to be agreeable, palatable, or satisfactory.	Satisfaction with various aspects of the innovation (e.g., content, complexity, comfort, delivery, and credibility)
Adoption	Intention, initial decision, or action to try or employ an innovation or evidence-based practice.	Uptake, utilization, initial implementation, intention to try
Appropriateness	Perceived fit, relevance, or compatibility of the innovation or evidence-based practice for a given practice setting, provider, or consumer; and/or perceived fit of the innovation or evidence-based practice to address a particular issue or problem.	Perceived fit, relevance. Compatibility, suitability, usefulness, practicability
Feasibility	Extent to which a new innovation or practice can be successfully used or carried out within a given agency or setting.<br>Reach + fidelity + dose = feasibility	Actual fit or utility, suitability for everyday use, practicability
Fidelity	Degree to which an intervention or implementation strategy was delivered as prescribed in the original protocol or as intended by program developers. May include multiple dimensions such as content, process, exposure, and dosage.	Delivered as intended, adherence, integrity, quality of program delivery
Implementation cost	Financial impact of an implementation effort. May include costs of treatment delivery, costs of the implementation strategy, and cost of using the service setting.	Marginal cost, cost-effectiveness, cost-benefit
Penetration	Extent to which an innovation or practice is integrated	Level of institutionalization, spread, service access
Sustainability	Extent to which a recently implemented practice is maintained and / or institutionalized within a service setting`s ongoing, stable operations.	Maintenance, continuation, durability; incorporation, integration, institutionalization, sustained use, routinization

Table 3

Stakeholder involvement screening terms for the identification of papers reporting on stakeholder involvement

**Stakeholder**	**Involvement**	**Other**
Stakeholder	Engage	Barriers
Community	Involve	Challenge
Patient	Partner-	Facilitators
Provider	Participat-	Interviews
Clinician	Collab-	Focus groups
Consumer	Co-	Surveys/quest-
End user	Input	Qualitative
Committee	Advisory/advi-	Dicussion
Board	Consult	Group
	-Led	Disseminate
	Group	Experience
	PAR	View
	Jury	Opinion
		Need
		Impact
		Outcome

Table 4

Stakeholder involvement categories from the GRIPP2 checklist and adapted research questions for this review

**Step 1**	**Research questions with focus on all identified implementation intervention studies (limited to general data on the manuscript)**		- What are the general characteristics of the publications: publication year, publishing journal, first author, article type?<br>- What are the general characteristics of the studies: setting, geographical locations (country), intervention, population, and (effectiveness & implementation) design?<br>- What is the total number of implementation intervention studies conducting stakeholder involvement (SI)?	
**Step 2**	**Research questions with focus on implementation intervention studies that performed a SE**			
**Category**	***GRIPP2 topic***	**GRIPP2 explanation**	***Data extraction topic for the EGM***	**Research question for the EGM data extraction**
**Theoretical information on SI**	***Theoretical underpinning***	Report the theoretical rationale and any theoretical influences relating to PPI in the study	***Theoretical gap in SI***	Was there a theoretical gap reported as a reason to conduct SI in the research study?
	Concepts and theory development	Report any conceptual models or influences used in the study	***Theory/model/framework for SI***	Was there a theory/model/framework used to guide SI and which?
	Aim	Report the aim of the study	***Aim/purpose of SI***	What is the aim of conducting SI in the research study according to the conceptualization of SI? (i.e. "*an iterative process of actively soliciting the knowledge, experience, judgment and values of stakeholders for the dual purposes of creating a shared understanding & making relevant, transparent and effective decisions*")?
**Stakeholder information**	***People involved***	Provide a description of patients, carers, and the public involved with the PPI activity in the study	***Stakeholders***	Who are the stakeholders that are engaged in the research project (7Ps)? (Concannon et al., 2012; Deverka et al., 2012)

				- Target population (who receives the intervention)<br>- Public/community and organisations<br>- Caregivers/family<br>- Healthcare stakeholders/institutions<br>- Payers/funders<br>- Purchasers<br>- (Local) government/leadership (policy makers)<br>- Product makers<br>- Other researchers (not part of the research team)<br>- Other
			Identification & recruitment	How were these stakeholders identified (and recruited) for involvement? (Concannon et al., 2019)
			Preparations	Where the stakeholders prepared in some form to be involved? (receive information, training, contracts, support,…) (Jo Brett et al., 2010)
			SI tasks	What are the specific tasks that the stakeholders were asked to do?
**Methodological information on SI**	***Stages of involvement***	Report on how PPI is used at different stages of the study	***Research phase***	In which research phase are stakeholders involved? Does the author specifically describe and explain the level or can this be derived from the text? (NIHR, 2021)<br>- Preparatory phase (~ CA)<br>- Baseline (T0)= before starting the intervention<br>- During the intervention<br>- Post intervention= at end of intervention or data collection when intervention has

				ended (e.g., looking at primary and secondary endpoints like QOL)<br>- Process evaluation= also post intervention data collection but not looking at primary/secondary endpoints but rather at intervention and its implementation
	Level or nature of involvement	Report the level or nature of PPI used at various stages of the study	***Level of involvement***	What is the level that the stakeholders are involved? Does the author specifically describe and explain the level or can this be derived from the text? (International association for public participation Australasia, 2018)<br>- Are stakeholders just informed about the research and its findings (informing/disseminating information= giving out information)<br>- Are stakeholders asked for their opinions (consultation= gathering information relevant to intervention implementation)<br>- Are stakeholders part of the research team and participate in the decision making/project management (collaboration/co-production= working closely with stakeholders, asking feedback and advice)<br>- Are stakeholders the ones who started and lead the research project (stakeholder led)
	Design	Provide a clear description of methods by which patients and the public were involved	***Methods***	Which methodological approach was applied to conduct SI? Does the author report any qualitative, quantitative or other methods ?
			Compensation for SI	What compensation did stakeholders receive for their involvement?

**Information on SI evaluation**			***Aim of SI evaluation***	Were SI efforts evaluated? What is the aim for evaluating the SI activity? (Popay et al., 2014)<br>- formative assessment (to improve involvement processes)<br>- process assessment (to identify factors affecting the involvement process)<br>- summative assessment (to demonstrate the outcomes of the involvement process)
	Outcomes of PPI	Report the results of PPI in the study, including both positive and negative outcomes	***Outcomes from SI***	Are there outcomes described from this SI? What are the (positive/negative) outcomes of SI? What came out of the involvement activity? (Ball et al., 2019; Staley, 2009)
	Impacts of PPI	Report the positive and negative impacts that PPI has had on the research, the individuals involved (including patients and researchers), and wider impacts and If applicable, comment on how well PPI impact was evaluated or measured in the study	***Impact of SI***	Are there impacts of involvement reported? What difference did involving stakeholders in the design and conduct of the research has made to the research process, the stakeholders, the research team, the wider setting/context and economic impact? (Ball et al., 2019; Staley, 2009)
	Qualitative/quantitative evidence of impact	If applicable, report the methods used to qualitatively/quantitatively explore the impact of PPI in the study	***Methods for (economic) evaluation***	What are the methods used for (economic) evaluation of SI?
	Economic assessment	If applicable, report the method used for an economic assessment of PPI and If applicable, discuss any aspects of the economic cost or benefit of PPI, particularly any suggestions for future economic modelling.		
	Robustness of measure	If applicable, report the rigour of the method used to capture or measure the impact of PPI	***Robustness/rigour***	Was the SI effort and its evaluation robust? Does the author state any evaluation of the robustness?

	Theory development	Report any conceptual or theoretical development in PPI that have emerged, evaluation of theoretical models, if any and Comment on any way your study adds to the theoretical development of PP	***TMF or any instrument development and evaluation***	If a TMF or other instrument for measurement was used or developed by the research team: was it evaluated?
	Measurement	If applicable, report all aspects of instrument development and testing		
	Context of PPI	Report the influence of any contextual factors that enabled or hindered the process or impact of PPI	***Contextual factors influencing SI***	What are contextual factors that enabled or hindered involvement? (Jo Brett et al., 2010)
	Process of PPI	Report the influence of any process factors, that enabled or hindered the impact of PPI	***Process factors influencing SI***	What are process factors that enabled or hindered involvement (how engagement was conducted)? (Jo Brett et al., 2010)
	Reflections/critic al perspective	Comment critically on the study, reflecting on the things that went well and those that did not, so that others can learn from this study		

Note. Data extraction was performed based on the guidance for reporting on involvement of patients and the public (GRIPP2) reporting checklist. (Staniszewska et al., 2017) The table presents the GRIPP2 checklist, with the topics to be reported in a paper listed on the left side. On the right side, it shows how we grouped into SI categories instead of paper sections and further adapted these topics for data extraction and the specific research questions we addressed during the process.

Table 5

Characteristics of all implementation studies included in step 1 (n=272) and SI studies in step 2 (n=241)

Characteristics of included articles	Studies step 1 (n; %)	Studies step 2 (n; %)
**Article type**		
- Protocol	195 (72%)	180 (75%)
- Original article	77 (28%)	61 (25%)
**Setting**		
- Community services (schools, churches, kindergarten,…)	99 (36%)	84 (35%)
- Hospital services	75 (28%)	66 (27%)
- Primary/ambulatory care	71 (26%)	65 (27%)
- Nursing homes	16 (6%)	15 (6%)
- Mental health clinics	11 (4%)	11 (5%)
**Country**		
- North America	108 (40%)	91 (38%)
- Europe	54 (20%)	49 (20%)
- Australia	40 (15%)	35 (14%)
- UK	26 (10%)	25 (10%)
- Africa	25 (9%)	23 (10%)
- Asia	15 (5%)	14 (6%)
- South America	4 (1%)	4 (2%)
**Design effectiveness evaluation**		
- Experimental design	239 (88%)	210 (87%)
- Quasi experimental design	30 (11%)	28 (12%)
- Unclear	3 (1%)	3 (1%)
**Hybrid design**		
- Hybrid I	27 (10%)	24
- Hybrid II	36 (13%)	32
- Hybrid III	16 (6%)	13
- Unclear/not reported	193 (71%)	172

Note. Tabulated characteristics of all implementation studies (n = 272) and of implementation studies reporting on stakeholder involvement (n= 241)

Table 6

Data on Stakeholder involvement summarized from the data extraction table

Studies reported on…	Total n= 241	
**Theoretical background on SI**	**N**	**%**
Aim/purpose of SI	196	81%
using a TMF	27	11%
theoretical gaps in SI	8	3%
**Stakeholder information**	**N**	**%**
Stakeholders involved	237	98%
Content of SI	228	95%
methods for identifying stakeholders	110	46%
Stakeholder preparation	9	4%
**Methodological information on SI**	**N**	**%**
level of SI	238	99%
methods for SI	238	99%
the research phase	217	90%
compensation for SI	27	11%
**Information on SI evaluation**	**N**	**%**
outcomes from SI	209	87%
impact of SI	154	64%
SI evaluation	10	4%
methods for SI (economic) evaluation	4	2%
contextual factors influencing SI	1	0,4%
process factors influencing SI	1	0,4%
robustness of SI/ SI evaluation	0	0%
evaluation of SI TMFs/measurement instruments used/developed	0	0%

Note. Abbreviations: TMF= theory, model, framework; SI= stakeholder involvement

Ball, S., Harshfield, A., Carpenter, A., Bertscher, A., & Marjanovic, S. (2019). *Patient and public involvement in research: Enabling meaningful contributions*. RAND Corporation. https://doi.org/10.7249/RR2678

Concannon, T. W., Grant, S., Welch, V., Petkovic, J., Selby, J., Crowe, S., Synnot, A., Greer-Smith, R., Mayo-Wilson, E., Tambor, E., & Tugwell, P. (2019). Practical Guidance for Involving Stakeholders in Health Research. *Journal of General Internal Medicine*, *34*(3), 458–463. https://doi.org/10.1007/s11606-018-4738-6

Concannon, T. W., Meissner, P., Grunbaum, J. A., McElwee, N., Guise, J.-M., Santa, J., Conway, P. H., Daudelin, D., Morrato, E. H., & Leslie, L. K. (2012). A new taxonomy for stakeholder engagement in patient-centered outcomes research. *Journal of General Internal Medicine*, *27*(8), 985–991. https://doi.org/10.1007/s11606-012-2037-1

Deverka, P. A., Lavallee, D. C., Desai, P. J., Esmail, L. C., Ramsey, S. D., Veenstra, D. L., & Tunis, S. R. (2012). Stakeholder participation in comparative effectiveness research: Defining a framework for effective engagement. *Journal of Comparative Effectiveness Research*, *1*(2), 181–194. https://doi.org/10.2217/cer.12.7

Gerke, D., Lewis, E., Prusaczyk, B., Hanley, C., Baumann, A., & Proctor, E. (2017). *Implementation Outcomes.*

International association for public participation Australasia. (2018). *IAP2 Public Participation Spectrum*. https://iap2.org.au/resources/spectrum/

Jo Brett, Staniszewska, S., Mockford, C., Seers, K., Herron-Marx, S., & Bayliss, H. (2010). *The PIRICOM study: A systematic review of the conceptualisation, measurement, impact and outcomes of patients and public involvement in health and social care research*. University of Warwick.

Mielke, J., Brunkert, T., Zúñiga, F., Simon, M., Zullig, L. L., & De Geest, S. (2022). Methodological approaches to study context in intervention implementation studies:

An evidence gap map. *BMC Medical Research Methodology*, *22*(1), 320. https://doi.org/10.1186/s12874-022-01772-w

NIHR. (2021). *Briefing notes for researchers: Public involvement in NHS, health and social care research*. NIHR. https://www.nihr.ac.uk/documents/briefing-notes-for-researchers-public-involvement-in-nhs-health-and-social-care-research/27371?pr=

Popay, J., Collins, M., & and with the PiiAF Study Group. (2014). *The public involvement impact assessment framework guidance.* Universities of Lancaster. https://piiaf.org.uk/

Proctor, E., Silmere, H., Raghavan, R., Hovmand, P., Aarons, G., Bunger, A., Griffey, R., & Hensley, M. (2011). Outcomes for implementation research: Conceptual distinctions, measurement challenges, and research agenda. *Administration and Policy in Mental Health*, *38*(2), 65–76. https://doi.org/10.1007/s10488-010-0319-7

Staley, K. (2009). *Exploring Impact: Public Involvement in NHS, Public Health and Social Care Research*. INVOLVE.

Staniszewska, S., Brett, J., Simera, I., Seers, K., Mockford, C., Goodlad, S., Altman, D. G., Moher, D., Barber, R., Denegri, S., Entwistle, A., Littlejohns, P., Morris, C., Suleman, R., Thomas, V., & Tysall, C. (2017). GRIPP2 reporting checklists: Tools to improve reporting of patient and public involvement in research. *Research Involvement and Engagement*, *3*(1), 13. https://doi.org/10.1186/s40900-017-0062-2
